# Epigenetic programming of pediatric high-grade glioma: Pushing beyond proof of concept to clinical benefit

**DOI:** 10.3389/fcell.2022.1089898

**Published:** 2022-12-14

**Authors:** Andrew Groves, Tabitha M. Cooney

**Affiliations:** ^1^ Division of Hematology/Oncology, University of Iowa Stead Family Children’s Hospital, Iowa City, IA, United States; ^2^ Dana Farber/Boston Children’s Cancer and Blood Disorder Center, Boston, MA, United States

**Keywords:** pediatric high grade glioma (pHGG), diffuse midline glioma (DMG), diffuse intrinsic pontine glioma (DIPG), DIPG (diffuse intrinsic pontine gliomas), oncohistone, epigenetics

## Abstract

Pediatric high-grade gliomas (pHGG) are a molecularly diverse group of malignancies, each incredibly aggressive and in dire need of treatment advancements. Genomic analysis has revolutionized our understanding of these tumors, identifying biologically relevant subgroups with differing canonical mutational profiles that vary based on tumor location and age. In particular, the discovery of recurrent histone H3 mutations (H3K27M in diffuse midline glioma, H3G34R/V in hemispheric pediatric high-grade gliomas) as unique “oncohistone” drivers revealed epigenetic dysregulation as a hallmark of pediatric high-grade gliomas oncogenesis. While reversing this signature through epigenetic programming has proven effective in several pre-clinical survival models, early results from pediatric high-grade gliomas clinical trials suggest that epigenetic modifier monotherapy will likely not provide long-term disease control. In this review we summarize the genetic, epigenetic, and cellular heterogeneity of pediatric high-grade gliomas, and highlight potential paths forward for epigenetic programming in this devastating disease.

## 1 Introduction

Central nervous system tumors are the most common solid tumor in children, and are the leading cause of cancer-related death in childhood (Ostrom et al., 2021). Pediatric high-grade gliomas (pHGG) are a biologically diverse group of tumors marked by an aggressive course with 5-year survival rates less than 30% (Ostrom et al., 2015). Numerous landmark studies over the last decade have dissected the molecular heterogeneity of pHGGs using exome, transcriptome, and methylome sequencing and outlined subgroups defined by key driver events which segregate in location and age-delimited fashion (Paugh et al., 2011; Schwartzentruber et al., 2012; Sturm et al., 2012). Despite these findings, standard of care treatment consisting of resection (when feasible), radiation ± alkylating chemotherapy has not changed in decades and offers little survival benefit (Cohen et al., 2011; Jakacki et al., 2016).

Epigenetic dysregulation as a mirror of disordered development is known to be a hallmark of pediatric cancer. For instance, although the global mutational burden of pediatric cancers is lower than adults, somatic mutations in epigenetic modifiers (like histone readers/writers/erasers and chromatin complexes) are the largest group of mutated genes identified in large sequencing studies (Grobner et al., 2018; Ma et al., 2018). This feature was also highlighted by the discovery of two specific pHGG mutational events in histone 3 (H3K27M present in ∼80% of diffuse midline gliomas and H3G34R/V present in ∼20% of hemispheric pHGG) occurring in hotspot histone residues (Schwartzentruber et al., 2012). While these oncohistones cannot yet be directly chemically targeted, a number of studies have investigated the effects of drugging other epigenetic regulators with several showing promise in pre-clinical models.

**FIGURE 1:**
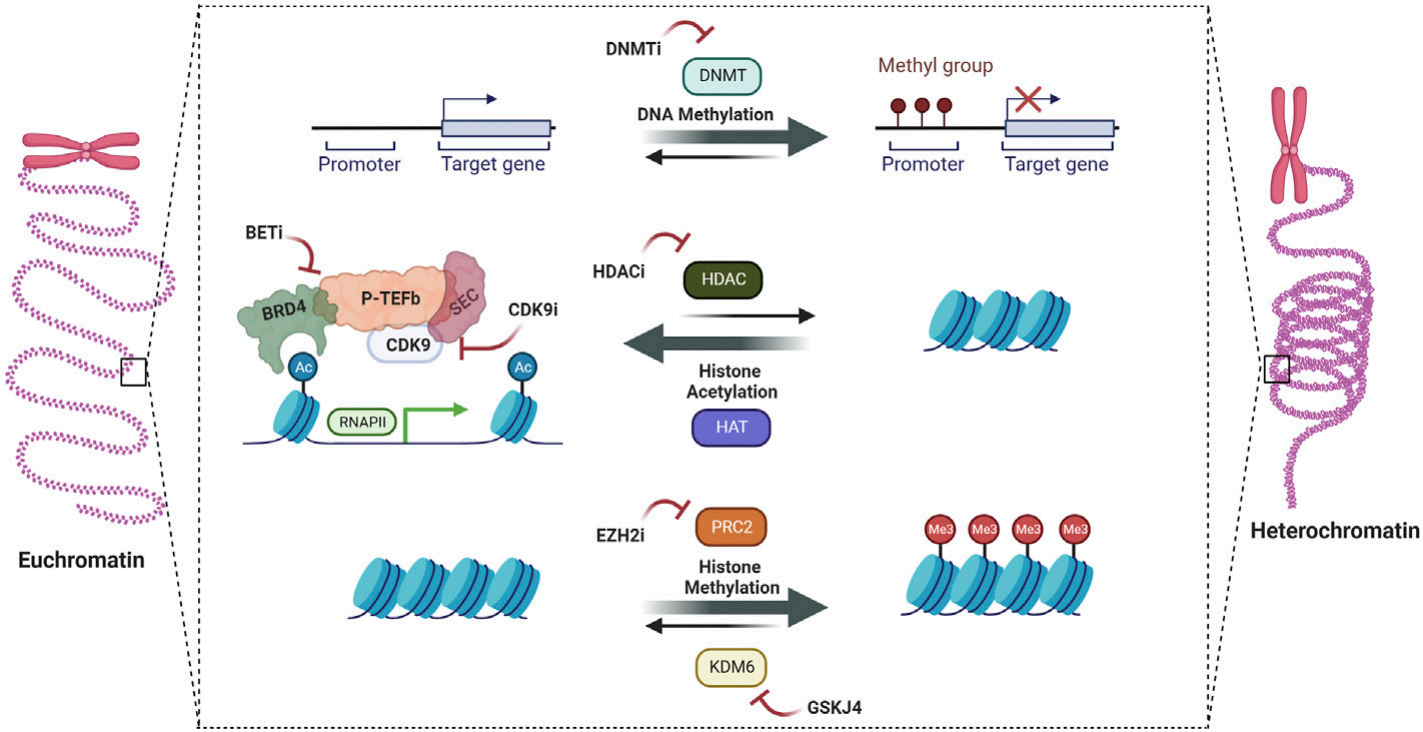
Epigenetic therapies in pediatric high-grade gliomas.

Despite the strong biologic rationale for this strategy, several questions remain. An important criticism levied is the narrow therapeutic window for targeting these epigenetic regulators given their importance in normal development and cell maintenance, which is only compounded by the need to achieve sufficiently high doses to cross the blood-brain barrier. In addition, resistance to epigenetic therapies is well documented in a variety of disease contexts as well as pHGG cell line models, suggesting that combination strategies will be needed. These questions and more will need to be addressed in ongoing and future clinical trials. In this review, we summarize the rationale, potential roadblocks, and translational opportunities for epigenetic programming in pHGG (Figure 1).

## 2 Developmental origins and molecular classification of pediatric high-grade glioma

Pediatric high-grade gliomas demonstrate a striking spatio-temporal and molecular distribution. For instance, H3K27M mutant tumors are predominantly seen in midline structures and in younger children, while H3G34R/V mutant tumors are restricted to the cerebral hemispheres and typically occur in older adolescence. These features suggest distinct cells of origin and a pathogenesis fundamentally linked to disordered neurodevelopmental processes. Indeed, an emerging theory of pediatric gliomagenesis is that cancer develops due to tumorigenic mutations or other transformational events occurring in cells of specific vulnerable transcriptional states, such as early neural or glial precursor cells. Several lines of evidence support this theory, including results from animal models and single-cell RNA sequencing of primary patient samples that have elegantly identified developmental hierarchies and candidate glioma stem cell’s (GSCs) of origin for pHGG subtypes that reflect intrinsic tumor heterogeneity. This work, in addition to large-scale genomic and epigenomic profiling efforts, led to the progressive reclassification of pHGG subtypes which was recently summarized in the fifth edition of the WHO Classification of Tumors of the CNS under the category of “Pediatric-type diffuse high-grade gliomas” (Louis et al., 2021).

### 2.1 Diffuse midline glioma, H3 K27-altered

Diffuse midline gliomas (DMG), H3 K27-altered was first outlined as a diagnosis in the 2016 CNS WHO classification and was defined by the presence of histone H3 K27M mutation, diffuse growth pattern, and midline location (Louis et al., 2016). This category included tumors previously called diffuse intrinsic pontine glioma (DIPG), of which ∼80% harbor the characteristic H3K27M mutation (Wu et al., 2014). These tumors are more commonly seen in pediatric patients (especially young children), have shared biologic properties (notably PRC2 inhibition and subsequent global loss of H3K27me3), and are associated with a dismal prognosis (Cooney et al., 2017). This diagnosis was updated in 2021 from “K27-mutated” to “K27-altered” based on the recognition that certain H3 WT DMGs have functionally equivalent PRC2 inhibition/H3K27me3 loss caused by EZHIP overexpression rather than H3K27 mutation (Jain et al., 2020a; Castel et al., 2020).

Histones are lysine and arginine rich proteins that condense DNA within eukaryotic nuclei into nucleosomes, the core unit of chromatin. Each histone has a highly conserved N-terminal amino acid “tail” that can undergo post-translational modifications (PTMs) which have profound effects on chromatin accessibility and transcription. Histone 3 (H3) is comprised of variants that include canonical H3.1 and variant H3.3, which are mutated in ∼15% and 85% of DMGs respectively. These heterozygous mutations canonically lead to a global decrease in the repressive H3K27 trimethylation mark through inhibition of PRC2 with concomitant increase in H3K27ac (Bender et al., 2013; Lewis et al., 2013). Interestingly, H3K27M mutations are found in only 5%–17% of the global histone pool, which can partially be explained by the fact that H3K27M is able to inhibit PRC2 in *trans* (Brown et al., 2014) The mechanism by which H3K27M induces these changes has been investigated by several studies, and proposed theories include H3K27M sequestration of PRC2, H3K27M-induction of defective spreading of PRC2-mediated H3K27me3 deposition, and transient H3K27M-PRC2 binding leading to irreversible inhibition of PRC2 catalytic function through an EZH2 conformational change (Justin et al., 2016; Stafford et al., 2018; Harutyunyan et al., 2019).

Regarding developmental origins, early studies identified a population of Nestin/SOX2/Olig2 positive stem cells in the human and murine developing brainstem as a candidate cell of origin (Monje et al., 2011). Later studies used single-cell RNA sequencing (scRNAseq) to dissect cellular heterogeneity in primary tumors, and described a large population of cycling stem cell-like oligodendroglial precursor cells (OPCs) in addition to more differentiated astrocytic (AC) and oligodendroglial (OC) cells (Filbin et al., 2018).

### 2.2 Diffuse hemispheric glioma, H3 G34-mutant

Approximately 15%–20% of hemispheric pHGGs harbor an alternate H3.3 mutation of glycine-to-arginine (or valine) in residue 34 (Sturm et al., 2012; Mackay et al., 2017). These tumors occur in adolescents/young adults (median age 15 years), and are similarly aggressive with a median overall survival of 18 months. Molecularly these tumors form a distinct cluster by DNA methylation profiling, and frequently have co-occurring mutations in TP53 and ATRX (Korshunov et al., 2016). Unlike H3K27, H3G34 is not subject to post-translational modifications but instead the H3G34R/V mutation has been found to inhibit SETD2-mediated deposition of H3K36me3 (Zhang et al., 2017a). Unlike K27-altered DMGs, the effects of H3G34R/V are only seen in *cis* (e.g., only mutant nucleosomes have H3K36me3 loss) and accordingly global levels of H3K36me3 are not significantly effected. Other epigenetic effects of the H3G34R/V mutation include promotion of PRC2 activity that leads to increased H3K27me3 in mutant histones (Jain et al., 2020b).

Studies using single-cell RNA sequencing and other epigenomic techniques have demonstrated that H3G34R/V mutant tumors express a primarily neuronal transcriptional profile with GSX2+ interneuron progenitors being the likely cell of origin (Chen et al., 2020). ScRNA-seq identified that unlike H3K27M DMG, H3G34R/V mutant tumors primarily express neuronal and astrocytic identities with absence of oligodendroglial programs. Interestingly, functional studies using human fetal neural stem cells recapitulated the observed locoregional incidence of oncohistone mutations by showing that induction of H3K27M or H3G34R/V in these stem cells has differential effects in forebrain vs. hindbrain-derived stem cells (Bressan et al., 2021).

### 2.3 Diffuse pediatric-type high-grade glioma, H3-wildtype and IDH-wildtype

Histone 3 and IDH wildtype pHGG are a heterogeneous group of tumors that have been molecularly classified into three subgroups (Korshunov et al., 2017; Mackay et al., 2017). Significant biological and clinical variability has made studying the developmental origins and functional effects of epigenetic programming on H3/IDH-WT pHGG tumors more challenging. More work is desperately needed for this patient population, and will hopefully be spurred on by the development of more pre-clinical models (He et al., 2021).

### 2.4 Infant-type hemispheric glioma

Infant brain tumors (definition varies, but in general age <3–5) have unique biological features, and treatment poses unique challenges due to the inability to offer radiation secondary to associated devastating long-term sequelae such as intellectual disability, endocrine dysfunction, and secondary neoplasms (Walter et al., 1999). Infantile high-grade gliomas have a significantly improved prognosis compared to HGG occurring in older children, and chemotherapy with protocols such as Baby POG have been shown to be an effective radiation-sparing treatment approach (Duffner et al., 1993). The clinical behavior of infantile HGGs suggests differing biological origins and molecular drivers, which was confirmed by advanced genomic profiling that showed a high frequency of pathogenic gene fusions in ALK, NTRK1/2/3, ROS1, or MET (Guerreiro Stucklin et al., 2019; Clarke et al., 2020). Subsequent research has not been focused on epigenetic programming, but rather utilizing targeted inhibitors to disrupt the relevant oncogenic fusion. Encouraging results have been seen in several case reports/series, and an ongoing phase 1 study will test the efficacy of Larotrectinib in combination with standard of care chemotherapy (BabyPOG or HIT-SKK) in newly diagnosed infantile HGG with NTRK fusions (Drilon et al., 2017; Ziegler et al., 2018, NCT04655404).

## 3 Epigenetic programming and combination strategies

The identification of oncohistone mutations in pHGG led to the investigation of drugs targeting epigenetic regulators. Broadly speaking, these drugs work by altering DNA methylation patterns, histone post-translational modifications (PTM), or chromatin-remodeling complexes to reverse malignant expression signatures (Bates, 2020). Mechanistically, they are traditional small-molecule inhibitors targeting enzymes such as DNA methyltransferase, one of the many categories of histone PTM regulators including readers, writers, erasers, or chromatin remodeling complexes. Each category of epigenetic regulator has been investigated pre-clinically in pHGG, with a bias towards study in H3K27M-altered DMG in part due to the availability of patient-derived xenograft (PDX) models (Hermans and Hulleman, 2019). Below, we will summarize these studies as well as research into combination therapies and ongoing/completed clinical trials.

### 3.1 Histone deacetylase (HDAC) inhibitors

A landmark study published in 2016 performed a chemical screen in 14 patient-derived DMG cell lines, and found that the HDAC inhibitor panobinostat had potent effects *in-vitro* and in orthotopic xenograft models (Grasso et al., 2015). Of note, follow-up studies showed that extended treatment of genetic and orthotopic DMG mouse models at 10–20 mg/kg IP dosing was not tolerated, while reduced-dosed regimens were tolerated yet ineffective at prolonging survival (Hennika et al., 2017). The mechanism underlying HDAC inhibitor efficacy is not fully understood; however, studies have shown that it increases global H3K27 trimethylation and disrupts super-enhancer associated transcription through selective reduction of H3K27Ac (Nagaraja et al., 2017).

Several clinical trials of HDAC inhibitors in DMG have either completed or are ongoing (Table 1). The Children’s Oncology Group (COG) performed a phase I/II trial of vorinostat + radiation followed by maintenance vorinostat in children with DIPG. While vorinostat was well tolerated it did not have any effect on survival with 1-year EFS of 5.85% (Su et al., 2022) This negative result was likely multifactorial, with some contribution being limited CNS penetration (Hanson et al., 2013; Guntner et al., 2020). Panobinostat has been studied in a phase 1 trial through the Pediatric Brain Tumor Consortium (PBTC) for DIPG, with results having recently being published (Cooney et al., 2018; Monje et al., 2022). In the first stratum of patients, the maximal tolerated dose on a schedule of 3 weeks on/1 week off M/W/F was lower than expected at 10 mg/m2/dose with dose-limiting toxicities (DLT) being primarily hematologic. A second stratum was subsequently opened with an alternating week dosing schedule, and while dose level 2 was established as tolerated there was no significant difference in PFS or OS compared to historical controls. In order to bypass concerns of sub-optimal CNS penetration, an alternative strategy was to deliver a water-soluble formulation of panobinostat though direct convection-enhanced delivery (CED) in a recently completed Pacific Pediatric Neuro-Oncology Consortium (PNOC) phase 1/2 clinical trial for children with DIPG (Singleton et al., 2018; NCT03566199).

**TABLE 1:** Pre-clinical epigenetic targeting strategies and stage of clinical development.

Primary category	Drug/Drugs	Combination category	Drug/Drugs	Pediatric DMG clinical trials^a^
HDAC	Vorinostat	N/A	N/A	NCT01189266 (COG ACNS0927)
		mTOR	Temsirolimus	NCT024220613
	Panobinostat	N/A	N/A	NCT02717455 (PBTC-047), NCT04264143, NCT03566199 (MTX110)
		Proteasome	Marizomib	NCT04341311
		BET	JQ1	
		CDK7	THZ1	
		AXL	BGB324	
		FACT	CBL0137	NCT04870944 (CBL0137 monotherapy)
		DNMT	5-azacitidine	
		BPTF	NA	
	Entinostat	KDM1A/LSD1	Compound 7,Corin	
HDAC/PI3K	Fimepinostat	N/A	N/A	NCT03893487 (PNOC016)
JMJD3	GSK-J4	N/A	N/A	
KDM1A/LSD1	GSK-LSD1	NK cell infusion	N/A	
EZH2	EPZ6438/Tazemetostat	N/A	N/A	
		BET	JQ1	
BET	JQ1, BMS-986378	NA	NA	NCT03936465
		CBP	ICG-001	
		Notch	MRK-003	
DNMT	5-azacitidine	NA	NA	
BMI-1	PTC028, PTC596	NA	NA	NCT03605550
		BH3	ABT263, obatoclax	
BPTF	NA	NA	NA	
SEC, CDK9	Atuveciclib, AZD4573	NA	NA	

^a^ Source: Clinicaltrials.gov. HDAC, histone deacetylase; mTOR, mammalian target of rapamycin; BET, bromodomain and extraterminal proteins; CDK, Cyclin-dependent kinase; FACT, “facilitates chromatin transcription”; DNMT, DNA methyltransferase; BPTF, bromodomain PHD finger transcription factor; LSD1, lysine demethylase; PI3K, Phosphoinositide 3-kinase; JMJD3, jumonji domain-containing protein-3; NK, natural killer cell; EZH2, enhancer of zeste 2; CBP, CREB, binding protein; BM1-1, B lymphoma Mo-MLV insertion region 1 homolog; SEC, super elongation complex.

Pre-clinical work has long warned that resistance can develop quickly after HDAC inhibitor treatment, which has spurred on investigation into combination therapies. Synergistic effects have been shown with inhibition of the AXL pathway, the transcriptional master regulators BRD4 and CDK7, the lysine demethylase KDM1A/LSD1, DNA methylation, PI3K, FACT complex, and the proteasome to name a few (Nagaraja et al., 2017; Pal et al., 2018; Anastas et al., 2019; Krug et al., 2019; Lin et al., 2019; Meel et al., 2020; Ehteda et al., 2021). Two clinical trials investigating combination therapy with HDAC inhibitors are currently active: 1) a phase 1 trial with panobinostat and the proteasome inhibitor marizomib (NCT04341311) for children with DIPG, and 2) PNOC’s phase 1, target validation trial of the dual HDAC/PI3K inhibitor fimepinostat for children with DIPG, HGG, or medulloblastoma (NCT03893487).

### 3.2 Lysine demethylase inhibitors

Another rational strategy that has been investigated is reversing H3K27M-induced H3K27 trimethylation loss through inhibition of the H3K27-specific lysine demethylase JMJD3. Pre-clinical studies showed the JMJD3 inhibitor GSK-J4 does indeed restore H3K27 methylation, demonstrates potent *in-vitro* and *in-vivo* activity, and synergizes with radiation (Hashizume et al., 2014; Katagi et al., 2019). Unfortunately, GSK-J4 has not yet been pushed forward into clinical development. Inhibition of the histone demethylase KDM1A/LSD1 has also been tested and shown to have modest efficacy as monotherapy, and synergizes with NK-cell immunotherapy and HDAC inhibitors (Anastas et al., 2019; Bailey et al., 2020).

### 3.3 Lysine methyltransferase inhibitors

Underscoring the complexity of DMG biology, residual PRC2 activity has been shown to be essential for tumor growth through H3K27me3 retention and subsequent transcriptional repression of critical tumor suppressors such as CDKN2A (Chan et al., 2013; Mohammad et al., 2017). Concordantly, treatment with EZH2 inhibitors reverses this signature and decreases tumor growth *in-vitro* and *in-vivo*. A recent study advises caution for this line of therapy by suggesting a tumor suppressive role of EZH2 using functional genomic gain- and loss-of-function studies in DMG mouse models (Dhar et al., 2022). Another exciting avenue for future study of EZH2 inhibition is as an adjuvant to immunotherapy. Specifically, recent studies have shown that EZH2 inhibition can increase tumor expression of the disialoganglioside GD2 and enhance efficacy of anti-GD2 therapies (Kailayangiri et al., 2019; Mabe et al., 2022). Given the efficacy of GD2-directed chimeric antigen receptor (CAR) T-cells in DMG preclinical research and promising early data from clinical trials, combination with EZH2 inhibition and other epigenetic therapies is a strategy worthy of further investigation (Mount et al., 2018; Majzner et al., 2022).

### 3.4 BET inhibitors, DNMT inhibitors, and other epigenetic regulators

Early genomic studies of DMG showed that H3K27M colocalizes with H3K27ac at sites of active transcription, and one strategy to target this aberrant signature is to disrupt the bromodomain and extra-terminal “reader” proteins critical for promoting malignant transcription (Nagaraja et al., 2017; Piunti et al., 2017). Several pre-clinical studies have demonstrated efficacy of treatment with the canonical BET inhibitor JQ1, with synergistic combinations being reported with the CBP inhibitor ICG-001, EZH2 inhibitor EPZ-6438 (tazemetostat), and γ-secretase inhibitor MRK-003 (Taylor et al., 2015; Zhang et al., 2017b; Wiese et al., 2020). A phase 1 trial is currently studying the CNS penetrant BET inhibitor BMS-986378 (CC-90010) in relapsed/refractory pediatric brain tumors (NCT03936465).

The DNA methyltransferase inhibitor azacytidine has been shown to amplify expression of pathologically expressed endogenous retrovirus (ERV) repeat genome elements, leading to induction of cellular interferon responses, immune activation, and tumor cell death (Krug et al., 2019). This effect was further amplified when combined with HDAC inhibitors. Inhibition of BMI-1, a core component of the PRC1 complex, has been shown to induce senescence in preclinical DMG models, and a CONNECT Consortium phase 1b trial of the BMI-1 inhibitor PTC596 in patients with DMG is currently underway (Balakrishnan et al., 2020; NCT03605550). Another newly described target is the NURF complex subunit BPTF, with shRNA knockdown decreasing pediatric HGG growth *in-vitro* through downregulation of MYC pathway targets (Green et al., 2020). Lastly, the super elongation complex (SEC), which incorporates CDK9-containining P-TEFb to promote RNA-Pol II dependent transcription, has also been shown to be a dependency that can be exploited using CDK9 inhibitors (Dahl et al., 2020).

## 4 Clinical outlook and future directions

Targeting epigenetic mechanisms remains an attractive, biologically-informed strategy in pHGG as detailed above; however, several translational limitations still need to be addressed. First and foremost is the difficulty penetrating the blood brain barrier, which is a hurdle all CNS therapeutics face. Optimizing target validation efforts in early phase clinical trials will allow for efficient determination of pharmacodynamic (PD) endpoints, improving chances of therapeutic success and saving resources (Kinders et al., 2007; Kummar et al., 2007).

In a related vein are questions related to the therapeutic window of epigenetic modifiers given their importance in normal biologic processes. These limitations compound one another since doses needed to achieve therapeutically relevant concentrations in the tumor may be above the maximal tolerated dose for non-CNS tissues such as hematopoietic progenitor cells. Local drug delivery strategies continue to develop in an effort to optimize intra-tumoral delivery while minimizing potentially toxic systemic effects.

Next is a more fundamental concern regarding the clinical efficacy of epigenetic therapies in human cancers based on the finding that many cancers can develop relatively rapid resistance mechanisms, and the heretofore limited success in adult cancers to a few select hematologic malignancies (tazemetostat for epitheliod sarcoma being the one exception for solid tumors). Future work in pHGG will need to address these concerns, with a continued focus on combination therapies to combat resistance and lower the effective therapeutic dose. Combining epigenetic modifiers and immunotherapy is especially attractive given the positive preliminary results from GD2-CAR T and oncolytic virus trials (Gallego Perez-Larraya et al., 2022; Majzner et al., 2022).

Finally, as HGG trial design becomes more complex and multi-modal, comprehensively evaluating local and systemic biomarkers within said trials will be crucial to illuminate signal/noise. Our therapeutic strategies are evolving to match the biologic complexity of these tumors and investigators must do what they can to critically understand our future successes and failures.

## 5 Conclusion

Despite exponential growth in our understanding of the mechanisms that drive pHGGs, patient outcomes remain unacceptably poor. Epigenetic modifiers are worthy of continued clinical investigation given the stalled developmental roots of pHGG oncogenesis and the growing wealth of pre-clinical evidence. Continued collaboration between researchers, clinicians, and industry will be required to combat translational limitations and establish efficacy for epigenetic programming in this devastating disease.
